# Interactions among Natural Active Ingredients to Improve the Efficiency of Rumen Fermentation In Vitro

**DOI:** 10.3390/ani11051205

**Published:** 2021-04-22

**Authors:** Rokia Temmar, María Rodríguez-Prado, Gwenael Forgeard, Cécile Rougier, Sergio Calsamiglia

**Affiliations:** 1 Animal Nutrition and Welfare Serice, Departament de Ciència Animal i dels Aliments, Universitat Autònoma de Barcelona, 08193 Bellaterra, Spain; temmarrokia@gmail.com (R.T.); maria.rodriguez.prado@uab.cat (M.R.-P.); 2 TECHNA France Nutrition, BP 10-44220 Couëron, France; gwenael_forgeard@techna.fr (G.F.); cecile_rougier@techna.fr (C.R.)

**Keywords:** essential oils, mixtures, synergy, rumen microbial fermentation, dairy cow

## Abstract

**Simple Summary:**

Despite the high number of products based on essential oils (EO) mixtures as feed additives for dairy cattle on the market, few studies have been specifically designed to explore possible additive, synergistic or antagonistic effects. This study aims to explore additive, synergistic, or antagonistic effects of EO mixtures to improve feed efficiency in dairy cattle. A range of EO were screened individually or in combination for their effect on volatile fatty acids and ammonia nitrogen concentration. In general, EO used individually had small effects on rumen fermentation profile. However, when mixed, some EO resulted in synergistic effects while others had antagonistic effects.

**Abstract:**

Twelve essential oils (EO): Anise star, cassia, geraniol, lemongrass (LEM), limonene, thyme, tea tree, coriander (COR), capsicum, black pepper, turmeric and ginger (GIN), in Experiment 1 at three doses; and different combinations of LEM, COR and GIN oils in Experiment 2, were evaluated in in vitro batch microbial fermentation using ruminal fluid from four dairy cows fed a 50:50 forage: concentrate diet. In experiment 1, LEM tended to increase the propionate proportion and tended to decrease the acetate to propionate ratio. Anise star, COR, and thyme tended to increase butyrate proportion. Capsicum, COR, and thyme decreased ammonia-N concentration. In experiment 2, a synergy was observed between LEM and COR that resulted in an increase in total volatile fatty acids and propionate proportion, and a decrease in the acetate to propionate ratio. However, the addition of high doses of GIN to the mix had an antagonistic effect on the rumen fermentation profile of the LEM + COR mix. Careful selection and combination of these EO may result in useful mixtures with synergistic interactions to modulate rumen microbial fermentation profile.

## 1. Introduction

Ruminal microbial fermentation can be modulated with the use of additives, resulting in improved efficiency of energy and protein utilization [1]. Shifting ruminal fermentation profile toward more propionate and less acetate is more efficient and reduces the emission of methane [2]. For example, the addition of monensin increased propionate proportion, and decreased acetate and butyrate proportions, and therefore increase the efficiency of converting feed energy into energy in the acid end-products which are available for absorption [3,4,5]. However, since the ban on the use of antibiotics as growth promoters in animal feeds in the European Union in 2006 [6] the interest has focused on natural alternatives. Essential oils (EO) are the aromatic volatile fraction of plant secondary metabolites generally recognized as safe for human and animal consumption [7]. Essential oils are characterized by their active compounds included in two main chemical groups: terpenoids and phenylpropanoids [7,8]. Numerous studies have demonstrated the ability of EO and their main active components to shift rumen microbial fermentation profile [9,10,11]. Many plant extract additives in the market are based on combinations of several EO However, evidence of their possible additivity, synergistic or antagonistic effects is very limited, and only Fandiño et al. [12] recently reported a negative interaction of clove bud when mixed with thyme or oregano oils on ruminal microbial fermentation. Tserennadmid et al. [13] observed a synergy between α-pinene and limonene, two monoterpenes. Bassolé et al. [14] observed a synergy between the monoterpene linalool and the phenolic compound eugenol. In contrast, Gallucci et al. [15] observed an antagonistic effect between thymol and carvacrol, two phenolic compounds. However, all these studies on synergistic-antagonistic effects among EO have been conducted in the field of cosmetics and food processing [16,17]. Only few studies have been specifically designed to prove additivity, synergistic or antagonistic effects of EO on rumen microbial fermentation. Recently, Fandiño et al. [12] addressed this issue in high concentrate feedlot-type fermentation conditions and found an antagonistic effect when clove bud oil was mixed with tea-tree, thyme and (or) oregano oils. Our hypothesis was that the combination of EO at doses that would not have an effect when supplemented alone would have additive or synergistic effects when supplemented together. The objective of the present study is to explore additive, synergistic or antagonistic effects among EO to increase propionate and decrease acetate concentrations, and reduce the acetate to propionate ratio.

## 2. Materials and Methods 

An animal care and use statement was not needed in this study since the rumen fluid was obtained from an abattoir.

### 2.1. Experiment 1

#### 2.1.1. Experimental Procedures

A group of 12 EO at three different doses (Table 1) were selected based on previous studies [18,19,20], the Rumen Up project (https://www.abdn.ac.uk/research/rumen-up/ accessed on 18 September 2018), commercial availability, reasonable price and legal status in the EU. All EO were supplied by TECHNA France Nutrition (Couëron, France).

The experiment was conducted in in vitro batch fermentation conditions [21]. Rumen fluid was collected at a slaughterhouse from four dairy cows in each period, filtered through four layers of cheesecloth, mixed and transported to the laboratory in thermos with no headspace. The rumen fluid was pooled and added to a phosphate-bicarbonate buffer [22] in a 1:1 proportion, purged with N_2_, and adjusted to an initial pH of 6.6. The diet used was a 50:50 forage to concentrate dairy cow diet with 17.9% crude protein, 30% neutral detergent fiber, and 21% acid detergent fiber, dry matter (DM) basis, and consisted (DM basis) of alfalfa hay (50.3%), ground barley grain (19.1%), ground corn grain (19.1%), soybean meal (10.9%), and a vitamin-mineral dairy cow premix (0.6%). The diet was designed to meet or exceed nutrient recommendations for a Holstein cow (650 kg) producing 30 kg of milk [23]. Incubations were conducted in 110-mL polypropylene tubes containing 50 mL of culture fluid with 0.5 g of the diet cited above ground through a 1-mm screen (Cyclotec CT 293, Foss, Barcelona, Spain). Treatments included a negative control without additive (CTR), monensin as a positive control (MON; Sigma-Aldrich Chemical, St. Louis, MO, USA), a blank without additive nor diet, and each of the 12 EO with the diet at three doses: low, medium, and high (Table 1). All essential oils were dissolved in ethanol in different proportions to reach the appropriate dose, and a total of 0.2 mL was added to the culture fluid in each tube. The equivalent amount of ethanol (0.2 mL) was added to the control and the blank. Each treatment was used in triplicate at each dose, and fermentations were replicated in two independent periods. Anaerobiosis was ensured by the addition of N_2_ before sealing tubes with rubber stoppers. Incubations were conducted at 39 °C in a thermoregulated water bath.

#### 2.1.2. Sample Collection and Chemical Analyses

After 24 h of fermentation, final pH was measured with a pH meter (sensION+, HACH Company, Barcelona, Spain), and samples were collected for VFA (volatile fatty acids) and ammonia-N concentration analyses. Samples for VFA were analyzed by liquid chromatography. For that, 100 µL of sample was added to 50 µL of 2N HCl and shaken with a multivortex shaker for 5 s. Then, 1 mL of chloroform was added and shaken for 1 min again and centrifuged at 15,000× *g* for 5 min at 4 °C. The organic phase was collected and placed in a 2-mL Eppendorf containing 0.5 mL of 1M NaOH. Samples were shaken again for 3 min and centrifuged at 15,000× *g* for 5 min at 4 °C. The aqueous phase (0.4 mL) was collected, 50 µL of 2N HCl were added, shaken with a multivortex shaker for 5 min and centrifugated at 14,500× *g* during 5 min at 4 °C. The supernatant was placed in chromatographic glass vials before being injected for analysis. The HPLC system (HPLC 1100 series, Walldbroom, Germany) was composed of a quaternary pump, an automated injector, a column (Agilent, Sta. Clara, CA, USA) oven, and a UV detector. Branch-chained VFA were calculated as the sum of isobutyric and isovaleric acids. Ammonia-N concentration was analyzed as described by Chaney and Marbach [24], where 4 mL sample of fermentation fluid were acidified with 4 mL of 0.2N HCl and frozen. After thawing, samples were centrifuged at 15,000× *g* for 15 min at 7 °C, and the supernatant was analyzed by spectrophotometry (Libra S21, Biochrom Technology, Cambridge, UK).

#### 2.1.3. Statistical Analyses

The effect of different doses within each treatment compared with control was analyzed as a randomized block design using the MIXED procedure of SAS (v.9.4 SAS Institute, Inc., Cary, NC, USA). The dose was the fixed effect and the period (block) was the random effect. Treatment means were compared to the control using the Dunnett’s multiple comparison test [25], and were declared significant at *p* < 0.05, and 0.05 < *p* < 0.10 was considered a tendency. All means are reported as least squares means.

### 2.2. Experiment 2

#### 2.2.1. Experimental Procedures

**Figure 1:**
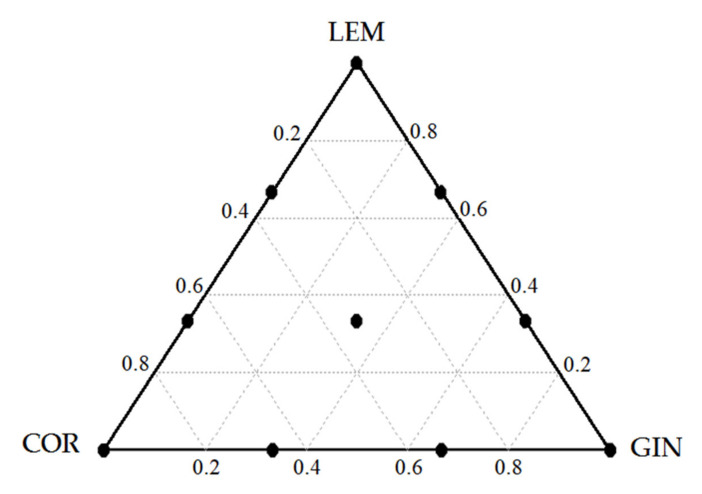
Simplex Centroid Design used to optimize the mixtures. The points correspond to the proportions of individual essential oils: lemongrass = LEM, coriander = COR, and ginger = GIN used in different combinations in this study.

Lemongrass (LEM), coriander (COR), and ginger (GIN) oils were chosen from the first experiment and used in different proportions according to the Simplex Centroid Design for experiments with mixtures ( [26], Figure 1). The three points at the vertices of the triangle correspond to the pure essential oils. The midpoints of the sides of the triangle correspond to 1:1 mixture of two essential oils. The center point of the triangle represents the three-component mixture in equal portions. Axial mixtures using different proportions were also investigated. These ten mixtures with different proportions of each component in the mix were prepared at a total of 75 mg/L for a low dose and a total of 150 mg/L for a high dose (Table 2). A control (CTR; diet without EO) and a blank (rumen fluid and buffer without feed nor EO) were included within each run. All EO were diluted in ethanol, and the control and the blank were also dosed with the equivalent amount of ethanol (0.2 mL). Fermentation conditions were the same as described in experiment 1. The incubations were conducted in triplicate and in two independent periods. At 24 h of fermentation, the final pH and samples for VFA and ammonia-N concentrations were collected and analyzed as described in the first experiment.

#### 2.2.2. Statistical Analyses

The statistical analysis was performed in two steps. The effect of dose (high and low) of each mixture of EO was analyzed using the MIXED procedure of SAS with the fixed effects of dose, treatment and their interaction, and the random effect of period. Treatments were compared with control using the Dunnett test. Results are reported as least squares means and significance was set at *p* < 0.05, and 0.05 < *p* < 0.10 was considered a tendency. The second analysis was for the Simplex Centroid Design using the R software from the R foundation for statistical computing (v. 4.0.2, 2020, https://www.R-project.org/ accessed on 2 February 2021). The analysis of such design is a multiple regression analysis without intercept [26]. The regression model included the three EO and their interactions as described below:

Y = a _lem_ × LEM + a _cor_ × COR + a _GIN_ × GIN + b _lem/cor_ × LEM × COR + b _lem/GIN_ × LEM × GIN +b _cor/GIN_ × COR × GIN + c _lem/cor/GIN_ × LEM × COR × GIN+ ε. (1)

where Y was the outcome (total VFA, A:P ratio, etc.); a, b, c were the regression coefficient associated with corresponding term; LEM, COR, and GIN were the values of the factor for the low and high doses (LEM + COR + GIN = 150 mg/L); and ε was the residual error. Then, the sum of squares of the prediction error (PRESS) was calculated. For each fitted value, PRESS is obtained by deleting the i_th_ observation from the data set, estimating the regression equation from the remaining n-1 observations, then using the fitted regression function to obtain the predicted value for the i_th_ observation. The predictive ability of the model was assessed with PRESS. In general, the smaller the PRESS value, the better the model’s predictive ability. The models adjusted in the second analysis was used to represent a contour plot. The contour plot provides a 2-dimensional view where all points that have the same response are connected by a line of constant responses.

## 3. Results

### 3.1. Experiment 1 (Individual Essential Oils)

In general, the majority of EO screened in this experiment and monensin did not modify the rumen fermentation profile (Supplementary Table S1–S6). The average concentration for total VFA was 74.0 ± 7.13. mM, for acetate proportion was 51.5 ± 1.92 mol/100 mol, for propionate proportion was 17.38 ± 1.58 mol/100 mol, for butyrate proportion was 20.0 ± 2.72 mol/100 mol, and for the A:P ratio was 3.07 ± 0.43. Total VFA concentration tended to increase only with the low dose of thyme oil. None of the doses of EO tested affected the acetate molar proportion. Only lemongrass oil at low dose tended to increase propionate proportion and tended to decrease the A:P ratio. Thyme oil at medium dose increased butyrate molar proportion. Coriander oil at low and high doses, and anise star oil at high dose tended to increase butyrate molar proportion. No effect of EO was observed on branch-chained VFA molar proportion. The effect of EO on ammonia-N is reported in Table 3. Capsicum, coriander, and thyme oils decreased ammonia-N concentration at low, medium, and high doses. Limonene tended to decrease ammonia-N concentration at medium and high doses. Cassia oil also tended to decrease ammonia-N concentration at high dose. However, geraniol increased ammonia-N concentration at low, medium, and high doses, and cassia oil tended to increase ammonia-N concentration at low and medium doses.

### 3.2. Experiment 2 (Mixtures of Essential Oils)

Results of the analysis of variance which consisted of comparing the different treatments versus the control in the case of the low dose, showed no difference between the treatments and the control (data not shown). Therefore, the Simplex Centroid Design analysis was not applied on low dose treatments and will not be discussed further.

**Figure 2:**
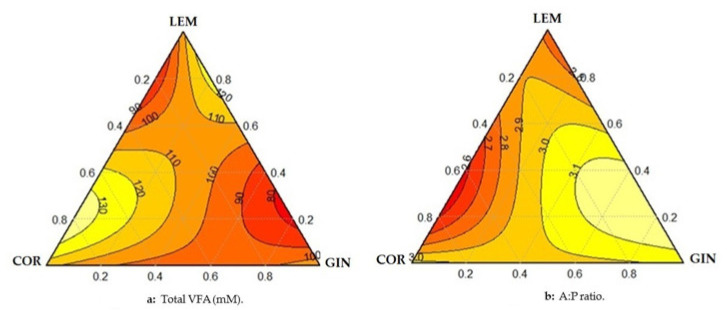
The two-dimensional contour plots for (**a**) total volatile fatty acids (VFA, mM) and (**b**) the acetate to propionate (A:P) ratio. The three vertices of the triangle represent individual essential oils: lemongrass = LEM, coriander = COR, and ginger = GIN. The contour plot provides a 2-dimensional view where all points that have the same response are connected by a line of constant responses.

Results from the analysis of variance for the high dose used in this study of the control treatment and the Simplex Centroid Design treatments for total and individual VFA and ammonia-N are presented in Table 4, Table 5 represents the coefficient estimates and statistics values when using the interaction model of the Simplex Centroid Design treatments. Contour plot for total VFA and A:P ratio is presented in Figure 2a,b, respectively.

The sum of the squares of the prediction error (PRESS) was low for all the outcomes (Table 5), which reflects the fit of the model used in this analysis. This precision of prediction indicates that the model represented correctly the response surface. Also, the high value of the R^2^ for all the outcomes (average R^2^ = 0.98) indicates that the model was adequate for the analysis of the response surface.

Within the Simplex Centroid Design treatments, the three EO used individually increased total VFA, where the highest value was observed in COR followed by LEM and GIN. The significant coefficient of total VFA (Table 5) for the mix containing equal proportion of LEM and COR indicated that the two EO were synergistic in increasing total VFA concentration. This synergism is also illustrated in the left part of Figure 2a where the maximum of total VFA was observed when the mixture is formed by LEM and COR in a range of 65–90% with a maximum of 90% for the COR and completed with 10% of LEM. Figure 2a also shows that increasing the proportion of GIN in the mixture decreased total VFA concentration, and, therefore, has an antagonistic effect when mixed with LEM and COR.

The three EO used individually decreased acetate, tended to decrease the A:P ratio, and increased propionate and butyrate molar proportions (Table 5). The mix containing equal proportions of LEM and COR resulted in the lowest A:P ratio and increased the propionate molar proportion (Table 5), suggesting that LEM and COR were synergistic in decreasing the A:P ratio and increasing propionate molar proportion. Figure 2b shows that the lowest A:P ratio was observed when the mixture is formed by LEM and COR in a range of 55–80% with a maximum of 80% for the COR and completed with 20% of LEM. In contrast, the addition of high doses of GIN increased the A:P ratio suggesting an antagonistic interaction of GIN versus LEM+COR. There were no synergies among the three EO on ammonia-N concentration. The lower value of ammonia-N was observed with COR used alone (Table 5). 

## 4. Discussion

### 4.1. Effect of Phenolic Compounds (Experiment 1)

The EO screened in this study can be classified according to their main active compound as phenolic compounds (anis star, black pepper, capsicum, cassia, ginger, thyme, and turmeric oils) and monoterpenes (coriander, geraniol, lemongrass, limonene, and tea tree). Both phenolic and monoterpene compounds have exhibited effective antimicrobial activity in vitro [7,27]. Phenolic compounds have been shown to have antimicrobial activity due to the presence of a hydroxyl group in the phenolic structure [17]. These molecules have a wide spectrum of activity against Gram-positive and Gram-negative bacteria [8,28]. However, in our study, the addition of phenolic compounds at low, medium, or high doses did not improve rumen fermentation profile in the desired direction (increasing propionate and decreasing acetate and the A:P ratio) except for thyme oil which tended to increase total VFA and butyrate proportion, and anise star oil that had a small effect only on butyrate molar proportion. Our results agree with previous studies that used thyme oil at doses ranging between our low and medium doses (at 100 or 200 mg/L) or its main active compound (thymol at 200 mg/L) in in vitro dairy cow rumen microbial fermentation conditions [20,27]. However, Castillejos et al. [9] used thymol at higher doses (500 and 5000 mg/L) and observed a decrease in total VFA and an increase in the A:P ratio, but such an effect is not beneficial for improving the energy efficiency. Busquet et al. [29] used anise oil and its main active compound (anethol) at 300 and 3000 mg/L, and observed a decrease in total VFA, propionate, and acetate molar proportions. However, Chaves et al. [30] used anethol at low dose (20 mg/L) and did not find effect on rumen microbial profile. The results obtained from capsicum in this study agree with those of Busquet et al. [29] that used capsicum at different doses (3, 30, 300, and 3000 mg/L) and did not find effect on total VFA and the A:P ratio. Moreover, Busquet et al. [29] and Nanon et al. [31] tested ginger oil in vitro at similar or higher doses and did not find any effect. In this study, cassia oil did not affect total and individual VFA. Busquet et al. [29] used cinnamaldehyde, one of the main active compounds of cassia at 3000 mg/L and observed a decrease in total VFA and an increase in propionate molar proportion. The same active compound was used in another in vitro study at 200 mg/L and had no effect on total and individual VFA [27]. In this study, black pepper and turmeric did not have any effect on total and individual VFA. Moreover, we did not find any study that used black pepper and turmeric oils or their main active compound (piperine and curcumin) in in vitro dairy cow rumen microbial fermentation conditions to compare with. 

In addition to total and individual VFA, ammonia-N concentration was also measured, and was decreased by capsicum and thyme at low, medium, and high doses, and in cassia oil at high dose. Several studies reported that EO affect deamination and reduce ammonia-N concentration in the rumen [32,33,34]. The mechanism of action has been attributed to the effect of EO on bacterial groups that intervene in deamination, like hyper-ammonia-producing bacteria and/or species of the *Prevotella* genus [33,34]. In contrast, cassia oil dosed at low and medium doses increased the concentration of ammonia-N. This effect is not beneficial, because ammonia-N concentration represents a loss of dietary nitrogen and a source of environmental pollution [35].

### 4.2. Effect of Monoterpene Compounds (Experiment 1)

A second group of active components used in this study were monoterpenes, which are widespread constituents of essential oils [36] and also exhibit antimicrobial activity due to their alkyl group that may affect growth and energy metabolism of different microbial populations in the rumen [37,38]. Among the monoterpenes screened, only lemongrass oil tended to increase propionate and tended to decrease the A:P ratio. In contrast, Hristov et al. [20] used lemongrass in vitro at dose slightly higher to our low dose (80 vs. 100 mg/L) and observed a decrease in total VFA and acetate molar proportion. Another study that used lemongrass at doses ranging between 186 and 1491 mg/L in vitro but in this case, lemongrass had no effect on the rumen microbial fermentation profile [31]. Joch et al. [39] used citral, the main active compound of lemongrass at high dose (888 mg/L) and observed a decrease in total VFA and propionate proportion and an increase in acetate molar proportion and A:P ratio. The dose of lemongrass (80 mg/L) used in our study seems to be the more adequate to get a positive effect, because higher doses had no effect or negative effects on rumen fermentation profile. Limonene used in this screening did not improve rumen microbial fermentation at low, medium, and high doses. These results agree with those of Joch et al. [39] that used limonene at higher dose compared with us (844 mg/L) but results were the same, no effects on total and individual VFA. In contrast, Castillejos et al. [9] used limonene at doses similar or higher than ours (50, 500 and 5000 mg/L) and observed a decrease in total VFA and the A:P ratio. The tea tree oil used in this experiment did not modify the rumen fermentation profile. This result agrees with those of Hristov et al. [20] that used tea tree oil (100 mg/L) and no effect was observed. Busquet et al. [29] used tea tree oil at higher dose (3000 mg/L) but total VFA and propionate and acetate molar proportions decreased. We did not find references related to the use of coriander in vitro to modify dairy rumen fermentation profile. However, Joch et al. [39] reported that the use of linalool, one of the main active compound of coriander oil, in vitro at 870 mg/L decreased total VFA and propionate proportion. Geraniol oil used in this study did not show any effect on total and individual VFA at low, medium, and high doses. However, Joch et al. [40] used geraniol at 300, 600, and 900 mg/L, and at 300 mg/L observed a decrease in total VFA and acetate proportion, and an increase in butyrate proportion and the A:P ratio. 

Ammonia-N concentration was decreased by coriander, and limonene oils at low, medium, and high doses. As discussed previously, these effects have been attributed to the effect of EO on bacterial groups that intervene in deamination, like hyper-ammonia-producing bacteria and/or species of the *Prevotella* genus [33,34]. In contrast, geraniol at low and medium doses increased the concentration of ammonia-N, which may reduce the efficiency of dietary nitrogen utilization and increase environmental pollution [35]. 

Overall, in the current study, EO supplemented individually did not improve the ruminal fermentation profile by increasing propionate and decreasing acetate and the A:P ratio.

### 4.3. Interaction among Different Essential Oils (Experiment 2)

Our hypothesis was that the combinations of EO at doses that would not have an effect alone would have additive or synergistic effects. For that, in the second experiment, three EO were selected. The selection criteria considered the potential interaction between different types of active compounds [14]. First, LEM containing citral as main active compound, is a monoterpene aldehyde that had positive effect on rumen fermentation in the first experiment. Coriander, containing linalool as main active compound was also selected as a monoterpene for its synergistic effect with phenolic compound [14]. Ginger was selected because its main active compound (gingerols) are phenolics. Although in the first experiment ginger oil did not influence rumen microbial fermentation, we hypothesized that a mix of phenol and monoterpene may result in synergistic effect, and a mix of two monoterpenes, LEM and COR, may also result in additive effect. The determination of interactions between the three EO selected was tested using the Simplex Centroid Design. Our results showed the existence of interactions. Synergy means that the effect of the mixture is higher than the effect of the individual compounds [17]. Significant coefficients (Table 5) for the two or the three mixed EO indicated that they interacted. Synergies were observed between LEM and COR, where the maximum for total VFA and the minimum for the A:P ratio were obtained by the mix of the two monoterpenes. In contrast, the addition of GIN to the mix decreased the total VFA and increased the A:P ratio suggesting an antagonistic effect of GIN versus LEM and COR on rumen fermentation. From Figure 2a,b, the optimum proportion of each EO in the mix to maximize the surface response for total VFA ranges between 65 and 90% with a majority of COR completed by LEM, and to minimize the surface response of the A:P ratio, the optimum proportion ranges between 55 and 80% with a majority of COR completed by LEM also. The matching zone between these values ranges between 65 and 80% with a maximum of 80% for COR and a minimum of 65% for LEM and must obey other criteria such as price and availability in the market. Results obtained from the analyses of the Simplex Centroid Design, to explore possible additive effect between two monoterpenes and a synergy between a monoterpene and phenol, did not support this hypothesis which was based on the study of Bassolé et al. [16] that observed an additive effect between monoterpenes and a synergy when using linalool and eugenol. In our case we observed a synergistic effect between two monoterpenes, LEM and COR, and an antagonistic effect when a phenol, GIN, was mixed with monoterpenes. Therefore, not all phenols can interact in the same way, and maybe the synergism found by Bassolé et al. [16] was specific for eugenol. In another study, Fandiño et al. [12] also reported antagonistic effect when clove bud oil was mixed with tea-tree, thyme, and (or) oregano oils. It is important to test the potential interactions between EO before mixing them to take advantage of the synergies and avoid the antagonistic effects.

## 5. Conclusions

In the first screening, the use of EO individually had small effects on rumen microbial fermentation. In the second experiment the use of the Simplex Centroid Design demonstrated a synergistic effect between LEM and COR to increase the total VFA concentration and propionate proportion, and to decrease the A:P ratio. However, an antagonistic effect was also observed when GIN oil was added at high doses to the LEM + COR mixture suggesting that only low doses should be used to optimize the ruminal fermentation. This study reveals the importance of taking into consideration the existence of positive and negative interactions among EO.

## Figures and Tables

**Table 1:** Essential oils, their main active compound, and different doses used in experiment 1.

Product	Active Compound and Purity (%)	Dose * (mg/L)		
		Low	Medium	High
Monensin	Monensin, 93	10		
Anise star	Trans-Anethol, 99	80	300	750
Black pepper	Piperine, 95	0.4	3	7.5
Capsicum	Capsaicin, 6	0.4	3	7.5
Cassia	Cinnamaldehyde, 75	80	300	750
Coriander	Linalool, 65	40	150	375
Geraniol	Geraniol, 84	80	300	750
Ginger	Gingerols, 63.2	10	40	150
Lemongrass	Citral, 75	80	300	750
Limonene	Limonene, 93	80	300	750
Tea tree	Terpinen-4-ol, 38.9	40	150	375
Thyme	Thymol, 47	80	300	750
Turmeric	Curcumin, 40	10	40	150

* The dose of each EO was calculated according to the % of purity of the main active compound.

**Table 2:** Determination of different proportions, combinations and doses of LEM, COR, and GIN used in experiment 2 according to the Simplex Centroid Design.

Treatments	Composition ^1^	%			Low Dose (mg/L)			High Dose (mg/L)		
		LEM	COR	GIN	lem	cor	gin	LEM	COR	GIN
T1	LEM	1.00			75.0			150.0		
T2	COR		1.00			75.0			150.0	
T3	GIN			1.00			75.0			150.0
T4	LEM + COR	0.50	0.50		37.5	37.5		75.0	75.0	
T5	LEM + GIN	0.50		0.50	37.5		37.5	75.0		75.0
T6	COR + GIN		0.50	0.50		37.5	37.5		75.0	75.0
T7	LEM + cor + gin	0.50	0.25	0.25	37.5	18.8	18.8	75.0	37.5	37.5
T8	lem + COR + gin	0.25	0.50	0.25	18.8	37.5	18.8	37.5	75.0	37.5
T9	Lem + cor + GIN	0.25	0.25	0.50	18.8	18.8	37.5	37.5	37.5	75.0
T10	LEM + COR + GIN	0.33	0.33	0.33	25.0	25.0	25.0	50.0	50.0	50.0

^1^ The treatments (LEM = lemongrass, COR = coriander, GIN = ginger) are written in upper case when they are the major essential oils (EO) in the mixture and in lower case when they are the minor essential oils in the mixture.

**Table 3:** Effect of essential oils on ammonia-N concentration (mg/100 mL) compared with control in in vitro rumen microbial fermentation of a 50:50 forage: concentrate diet.

Treatment	Control	Dose ^1^			SEM ^2^	*p*-Value
		Low	Medium	High		
Monensin	28.1	28.2			1.31	0.80
Anise star	28.1	29.2	28.5	27.5	2.60	0.56
Black pepper	28.1	26.4	26.7	26.3	0.96	0.48
Capsicum	28.1	25.2 *	24.3 *	25.5 *	1.56	0.02
Cassia	28.1	29.4 ^+^	30.8 ^+^	24.8 ^+^	1.74	0.09
Coriander	28.1	24.3 *	23.2 *	23.4 *	0.99	0.01
Geraniol	28.1	33.1 *	33.8 *	27.8	1.06	0.03
Ginger	28.1	29.7	31.0	29.9	1.50	0.14
Lemongrass	28.1	32.5	31.9	31.1	1.68	0.21
Limonene	28.1	28.9	27.6^+^	25.7 ^+^	2.20	0.07
Tea tree	28.1	25.9	27.7	28.8	1.75	0.12
Thyme	28.1	26.4 *	25.9 *	17.2 *	1.69	0.01
Turmeric	28.1	28.4	26.5	28.0	1.10	0.63

^1^ Doses for each EO are reported in Table 1; ^2^ Standard error of the mean. * Means within a row differ from control (*p* < 0.05), ^+^ means within a row differ from control (*p* < 0.10).

**Table 4:** Effect of mixtures of lemongrass (LEM), coriander (COR), and ginger (GIN) oils according to the Simplex Centroid Design on total and individual VFA, and ammonia-N concentration.

Treatment	Dose (mg/L)			TVFA ^1^	Acetate	Propionate	Butyrate	A:P ^2^	N-NH3
	LEM	COR	GIN	(mol/100 mol)	(%)	(%)	(%)		mg/100 mL
T1	150			109.0 *	52.4 *	19.0 *	24.6 *	2.77 ^+^	18.1
T2		150		114.0 *	54.1 *	18.1	24.1 *	3.02	17.9 *
T3			150	107.0 *	53.7 *	18.7	23.9 *	2.88 ^+^	18.7
T4	75	75		108.0 *	50.2 *	19.4 *	24.8 *	2.70 ^+^	18.7
T5	75		75	93.0	55.1 *	18.4	21.8 *	3.02	19.1 *
T6		75	75	91.0	56.8	18.2	21.9 *	2.97 ^+^	18.6
T7	75	38	38	100.0 *	54.8	17.6	18.0	3.06	20.4 *
T8	38	75	38	97.0	57.9	17.5	17.6	2.83 ^+^	20.0
T9	38	38	75	104.0 *	57.3	18.5	24.5 *	2.83 ^+^	19.8 *
T10	50	50	50	96.0	58.3	17.5	18.8	2.98 ^+^	18.8
Control	0	0	0	88.8	57.6	17.9	17.9	3.24	18.5
*p*‒Value				<0.01	<0.01	<0.01	<0.01	0.080	0.03
SEM ^3^				0.84	0.21	0.12	0.15	0.100	0.44

^1^ Total volatile fatty acids; ^2^ The acetate to propionate ratio; ^3^ Standard error of the mean; * Means within a column differ from control (*p* < 0.05); ^+^ Means within a column differ from control (*p* < 0.10).

**Table 5:** Results of the interaction model applied to the Simplex Centroid Design mixing lemongrass (LEM), coriander (COR), and ginger (GIN) oils on total and individual VFA and ammonia-N concentration. For each term of the model, the regression coefficients (Coef), their standard error (SEM), and the associated *p*-value are presented. The sum of squares of the prediction error is also shown (PRESS).

Treatment	TVFA ^1^			Acetate			Propionate			Butyrate			A:P ^2^			NNH3		
	Coef	SEM	*p*-Value	Coef	SEM	*p*-Value	Coef	SEM	*p*-Value	Coef	SEM	*p*-Value	Coef	SEM	*p*-Value	Coef	SEM	*p*-Value
LEM	111	4.54	<0.01	53.6	1.66	<0.01	18.9	1.62	<0.01	25.1	1.12	<0.01	2.74	0.31	<0.01	18.5	4.62	0.02
COR	114	4.54	<0.01	51.3	1.66	<0.01	18.8	1.62	<0.01	24.6	1.12	<0.01	3.04	0.31	<0.01	17.7	4.62	0.02
GIN	105	4.54	<0.01	55.0	1.66	<0.01	17.2	1.62	<0.01	23.3	1.12	<0.01	3.03	0.31	<0.01	18.6	4.62	0.02
LEM + COR	120	3.62	0.04	51.0	2.15	0.12	19.1	7.92	0.03	23.8	5.49	0.75	2.70	1.51	0.02	16.8	2.26	0.95
LEM + GIN	93	3.62	0.10	55.0	1.15	0.30	17.5	7.92	0.96	22.5	5.49	0.07	3.06	1.51	0.67	21.8	2.26	0.95
COR + GIN	97	3.62	0.14	54.1	2.15	0.78	17.0	7.92	0.91	23.0	5.49	0.20	2.83	1.51	0.87	22.3	2.26	0.89
LEM + COR + GIN	90	1.33	0.48	53.4	4.59	0.61	18.4	4.46	0.57	23.0	3.09	0.67	3.88	1.53	0.66	23.4	1.28	0.05
Statistical value																		
Linear	<0.01			<0.01			<0.01			<0.01			<0.01			<0.01		
Quadratic	<0.01			<0.01			<0.01			<0.01			<0.01			<0.01		
RSD ^3^	1.21			2.35			2.29			1.58			0.44			6.54		
R^2^	0.99			0.94			0.99			1.00			0.99			1.00		
Adjusted R^2^	0.99			1.00			0.98			1.00			0.98			0.89		
Predicted R^2^	0.97			1.00			0.97			0.99			0.96			0.80		
PRESS	0.07			0.07			0.03			1.23			0.03			0.09		

^1^ Total volatile fatty acids. ^2^ The acetate to propionate ratio; ^3^ Residual standard error.

## Data Availability

The data presented in this study are available on request from the corresponding author.

## Supplementary Materials

The following are available online at https://www.mdpi.com/article/10.3390/ani11051205/s1, Table S1: Effect of essential oils on total VFA concentration (mM) compared with control in in vitro rumen microbial fermentation of 50:50 forage: concentrate diet, Table S2: Effect of essential oils on acetate concentration (mM) compared with control in in vitro rumen microbial fermentation of 50:50 forage: concentrate diet. Table S3: Effect of essential oils on propionate concentration (mM) compared with control in in vitro rumen microbial fermentation of 50:50 forage: concentrate diet, Table S4: Effect of essential oils on butyrate concentration (mM) compared with control in in vitro rumen microbial fermentation of 50:50 forage: concentrate diet, Table S5: Effect of essential oils on BCVFA concentration (mM) compared with control in in vitro rumen microbial fermentation of 50:50 forage: concentrate diet, Table S6: Effect of essential oils on C2:C3 ratio compared with control in in vitro rumen microbial fermentation of 50:50 forage: concentrate diet.
