# Supplementary material for: Interactions among Natural Active Ingredients to Improve the Efficiency of Rumen Fermentation In Vitro
Source: Animals (Basel). 2021 Apr 22;11(5):1205. doi: 10.3390/ani11051205 (PMC8144957; doi:10.3390/ani11051205)
Supplement: Supplementary file 1 [file animals-11-01205-s001.zip › animals-1171591-supplementary.pdf]

Interactions Among Natural Active Ingredients to Improve the Efficiency of Rumen Fermentation *In Vitro*

Rokia Temmar, María Ercilda Rodríguez-Prado, Gwenael Forgeard, Cécil Rougier and Sergio Calsamiglia

Table S1. Effect of essential oils on total VFA concentration (m*M*) compared with control in *in vitro* rumen microbial fermentation of 50:50 forage: concentrate diet

Treatment	CTR	Dose (mg/L)			SEM	*p*-Value
		Low (0.4-80)	Med (3-300)	High (7.5-750)		
Anise	76.3	77.5	77.5	69.9	8.98	0.72
Capsicum	76.3	74.4	74.6	76.8	8.19	0.91
Cassia	76.3	79.7	66.8	54.9	11.9	0.25
Lemongrass	76.3	73.1	66.6	65.2	10.8	0.44
Geraniol	76.3	79.2	70.2	77.9	11.3	0.68
Ginger	76.3	76.3	78.0	77.6	6.56	0.89
Limonene	76.3	73.7	72.4	74.6	10.3	0.93
Coriander	76.3	76.2	76.3	72.9	7.90	0.85
Black pepper	76.3	78.1	78.4	80.1	7.12	0.85
Tea tree	76.3	72.0	78.3	75.5	8.47	0.66
Thyme	76.3	81.3	72.4	43.7	9.13	0.08
Turmeric	76.3	76.4	77.9	73.9	6.69	0.82

Table S2. Effect of essential oils on acetate concentration (mM) compared with control in in vitro rumen microbial fermentation of 50:50 forage: concentrate diet

Treatment	CTR	Dose (mg/L)			SEM	*p*-Value
		Low (0.4-80)	Med (3-300)	High (7.5-750)		
Anise	54.7	51.9	51.0	52.6	2.29	0.52
Capsicum	54.7	50.9	51.5	51.3	1.41	0.33
Cassia	54.7	51.6	50.7	57.0	3.87	0.64
Lemongrass	54.7	51.9	54.1	55.8	2.29	0.67
Geraniol	54.7	53.6	51.5	49.3	1.70	0.21
Ginger	54.7	49.7	50.9	50.0	2.19	0.44
Limonene	54.7	51.6	50.5	47.4	2.43	0.33
Coriander	54.7	49.9	49.9	49.2	2.05	0.34
Black pepper	54.7	50.4	51.4	52.1	1.96	0.52
Tea tree	54.7	48.8	51.9	50.6	2.31	0.43
Thyme	54.7	52.4	49.6	54.0	1.75	0.25
Turmeric	54.7	52.2	51.4	51.0	2.33	0.76

Table S3. Effect of essential oils on propionate concentration (m*M*) compared with control in *in vitro* rumen microbial fermentation of 50:50 forage: concentrate diet

Treatment	CTR	Dose (mg/L)			SEM	*p*-Value
		Low (0.4-80)	Med (3-300)	High (7.5-750)		
Anise	17.2	18.0	18.2	14.0	1.85	0.31
Capsicum	17.2	18.2	18.0	18.3	1.77	0.72
Cassia	17.2	17.8	18.0	17.4	2.61	0.97
Lemongrass	17.2	18.8	14.3	12.4	1.65	0.07
Geraniol	17.2	18.2	17.7	14.5	2.30	0.23
Ginger	17.2	17.6	18.3	18.5	1.83	0.65
Limonene	17.2	18.9	17.9	15.1	2.13	0.26
Coriander	17.2	18.1	18.6	17.2	2.08	0.71
Black pepper	17.2	18.1	18.2	17.5	2.00	0.83
Tea tree	17.2	16.3	18.0	18.4	1.62	0.57
Thyme	17.2	16.7	14.3	16.3	1.72	0.45
Turmeric	17.2	18.2	17.9	18.6	1.41	0.47

Table S4. Effect of essential oils on butyrate concentration (mM) compared with control in in vitro rumen microbial fermentation of 50:50 forage: concentrate diet

Treatment	CTR	Dose (mg/L)			SEM	*p*-Value
		Low (0.4-80)	Med (3-300)	High (7.5-750)		
Anise	19.1	17.5	20.0	24.7	1.30	0.09
Capsicum	19.1	19.4	19.2	19.1	0.66	0.98
Cassia	19.1	19.6	20.3	14,7	3.67	0.94
Lemongrass	19.1	18.8	24.4	24.7	2.04	0.20
Geraniol	19.1	19.3	20.2	24.7	1.40	0.13
Ginger	19.1	20.3	19.1	19.7	1.06	0.49
Limonene	19.1	18.3	20.3	26.1	1.75	0.11
Coriander	19.1	20.0	19.3	21.3	0.65	0.07
Black pepper	19.1	19.8	14.0	19.1	0.44	0.63
Tea tree	19.1	23.5	18.8	19.6	1.83	0.36
Thyme	19.1	19.2	24.5	19.2	0.92	0.03
Turmeric	19.1	19.8	18.9	15.0	0.89	0.77

Table S5. Effect of essential oils on BCVFA concentration (m*M*) compared with control in *in vitro* rumen microbial fermentation of 50:50 forage: concentrate diet

Treatment	CTR	Dose (mg/L)			SEM	*p*-Value
		Low (0.4-80)	Med (3-300)	High (7.5-750)		
Anise	6.27	8.03	7.88	5.42	3.18	0.48
Capsicum	6.27	9.03	8.93	8.95	1.97	0.56
Cassia	6.27	7.75	6.88	5.20	2.7	0.59
Lemongrass	6.27	7.66	3.90	3.56	2.47	0.63
Geraniol	6.27	5.95	6.79	7.03	3.66	0.86
Ginger	6.27	8.51	9.32	9.25	1.99	0.56
Limonene	6.27	8.39	7.88	7.71	2.21	0.67
Coriander	6.27	9.69	9.91	9.64	2.11	0.36
Black pepper	6.27	9.29	9.10	9.04	1.91	0.56
Tea tree	6.27	7.85	8.98	9.07	2.08	0.64
Thyme	6.27	9.37	8.67	6.51	2.02	0.49
Turmeric	6.27	7.36	9.39	8.49	2.29	0.79

Table S6. Effect of essential oils on C2:C3 ratio compared with control in *in vitro* rumen microbial fermentation of 50:50 forage: concentrate diet

Treatment	CTR	Dose (mg/L)			SEM	*p*-Value
		Low (0.4-80)	Med(3-300)	High (7.5-750)		
Anise	3.18	2.97	2.87	3.77	0.31	0.30
Capsicum	3.18	2.84	2.89	2.84	0.31	0.62
Cassia	3.18	2.96	2.8	3.39	0.39	0.54
Lemongrass	3.18	2.83	3.84	4.00	0.39	0.09
Geraniol	3.18	2.99	2.91	4.70	0.41	0.57
Ginger	3.18	2.87	2.84	2.75	0.38	0.64
Limonene	3.18	2.70	2.87	3.00	0.45	0.70
Coriander	3.18	2.80	2.74	2.95	0.42	0.71
Black pepper	3.18	2.80	2.80	3.00	0.41	0.79
Tea tree	3.18	3.00	2.93	2.70	0.33	0.71
Thyme	3.18	3.50	3.59	3.30	0.44	0.85
Turmeric	3.18	2.90	2.92	2.83	0.32	0.67
